# Pulsed radiofrequency for treatment of complex regional pain syndrome: A scoping review

**DOI:** 10.1016/j.inpm.2026.100771

**Published:** 2026-05-18

**Authors:** Kian Bagheri, Cara Wyant, Emily Brennan, Sarah Wade, Catherine Wagner, Alex Reid, Ameet Nagpal

**Affiliations:** a Honor Health Medical Center, Department of Physical Medicine and Rehabilitation, Scottsdale, AZ, 85258, USA; b Medical University of South Carolina Library, Charleston, SC, 29464, USA; c Campbell University School of Osteopathic Medicine Medical Library, Lillington, NC, 27546, USA; d Honor Health, Department of Graduate Medical Education, Scottsdale, AZ, 85258, USA; e Medical University of South Carolina, Department of Orthopaedics and Physical Medicine & Rehabilitation, Charleston, SC, 29464, USA

**Keywords:** Complex regional pain syndrome, CRPS, Pulsed radiofrequency, PRF, Chronic pain, Neuromodulation, Scoping review

## Abstract

**Background:**

Complex Regional Pain Syndrome (CRPS) is a debilitating chronic pain condition characterized by sensory, motor, and autonomic disturbances, often resistant to conventional therapies. Pulsed radiofrequency (PRF), a non-neurodestructive neuromodulation technique, has been described as a potential intervention for CRPS, though its clinical effectiveness remains uncertain due to limited evidence.

**Objective:**

To summarize the existing literature on PRF for the management of CRPS, including study designs, procedural characteristics, outcome measures, and reported safety considerations, and to identify knowledge gaps to guide future research.

**Methods:**

A comprehensive literature search was conducted across PubMed, Scopus, APA PsycINFO, CINAHL, and SPORTDiscus from inception to October 31, 2025. Eligible studies included randomized controlled trials, observational studies, case series, and case reports reporting on PRF for CRPS. Data were charted for study characteristics, intervention details, outcome domains, and adverse events. No formal risk of bias assessment or meta-analysis was performed, consistent with scoping review methodology.

**Results:**

Eleven studies involving a total of 43 patients were included, consisting of case reports, case series, and small retrospective cohorts, with no randomized controlled trials identified. PRF targeting sympathetic structures, dorsal root ganglia, or peripheral nerves was generally associated with reductions in pain intensity across short-to-mid-term follow-up, and no serious adverse events were reported.

**Conclusion:**

PRF appears to be a safe, minimally invasive intervention with potential benefit for pain reduction in CRPS. However, the current evidence is limited by small sample sizes, heterogeneity, and inconsistent reporting, highlighting the need for well-designed randomized controlled trials to clarify its role in CRPS management.

## 1 Introduction

Complex Regional Pain Syndrome (CRPS) is a challenging and often debilitating chronic pain condition characterized by severe, persistent regional pain that is disproportionate to any initial injury [1,2]. It is frequently accompanied by a constellation of sensory abnormalities, motor dysfunction, autonomic disturbances, and trophic changes affecting the skin, muscles, and bones. CRPS is broadly classified into Type I, which occurs without identifiable nerve injury, and Type II, which involves a confirmed nerve lesion [1]. The pathophysiology of CRPS is complex and multifactorial, involving peripheral and central sensitization, neurogenic inflammation, autonomic dysregulation, and maladaptive cortical reorganization [3]. These mechanisms contribute to the heterogeneity of clinical presentation and often make management difficult [4].

Traditional therapeutic approaches for CRPS encompass a multidisciplinary framework, including pharmacologic treatments such as analgesics, corticosteroids, and neuropathic agents; physical and occupational therapy aimed at improving function and preventing disuse; sympathetic nerve blocks; and psychological interventions [5]. Among available interventions, neuromodulation, particularly spinal cord stimulation (SCS) and dorsal root ganglion stimulation (DRG-S), has the most robust clinical evidence supporting its efficacy for pain reduction and functional improvement in refractory CRPS [6,7]. Despite these efforts, many patients experience refractory pain and disability, underscoring the need for innovative and effective treatment modalities [8,9].

Pulsed radiofrequency treatment (PRF) has emerged as a minimally invasive neuromodulatory technique that applies brief bursts of high-frequency electrical current to neural structures [10]. Unlike continuous radiofrequency ablation, PRF does not produce significant thermal injury, thus preserving nerve integrity while modulating pain pathways [11]. PRF has been utilized in the management of various chronic pain conditions, including CRPS, with reported benefits in pain reduction and functional improvement [12]. Anatomical targets for PRF in CRPS include the dorsal root ganglion, sympathetic chain, and peripheral nerves, though protocols vary widely in terms of parameters such as voltage, pulse duration, and treatment cycles [12].

Despite increasing clinical adoption, the evidence supporting PRF for CRPS remains fragmented, with studies demonstrating variable methodological quality, heterogeneous patient populations, inconsistent application parameters, and diverse outcome measures [13]. The lack of standardized reporting further complicates the interpretation and generalizability of results [14]. A scoping review is thus warranted to systematically map the available evidence, describe study characteristics and intervention techniques, summarize outcome domains and reporting patterns, and identify knowledge gaps to guide future research.

## 2 Methods

### 2.1 Search strategy

This scoping review was conducted following the Preferred Reporting Items for Systematic Reviews and Meta-Analyses extension for Scoping Reviews (PRISMA-ScR) [15]. To identify studies for inclusion in this scoping review, two health sciences librarians developed detailed search strategies in PubMed, Scopus, CINAHL, PsycINFO, and SPORTDiscus. ClinicalTrials.gov (U.S. National Library of Medicine, National Center for Biotechnology Information) and the International Clinical Trials Registry Platform (ICTRP) (World Health Organization) were also searched but retrieved zero results. The databases were searched from inception through October 31, 2025. English language filters were applied. The search strategies used a combination of subject headings (when available) and keywords for the concepts of complex regional pain syndromes and pulsed radiofrequency. The PubMed search strategy was modified for the other databases, replacing MeSH terms with appropriate subject headings when available and retaining similar keywords. The search strategies were peer-reviewed by a health sciences librarian using a modified PRESS checklist (cite PRESS). The literature search was reported according to PRISMA-S, an extension of the PRISMA Statement for reporting literature searches. The full, reproducible search strategies for all included databases are provided in the Appendix/Supplementary Materials section.

### 2.2 Eligibility criteria and study selection

Eligible studies included those that enrolled patients of any age diagnosed with CRPS type I or II, based on recognized diagnostic criteria such as the Budapest criteria or International Association for the Study of Pain (IASP) criteria. The intervention of interest must be PRF targeting any relevant anatomical site, including the dorsal root ganglion (DRG), sympathetic chain, or peripheral nerves. Studies reporting any clinical outcomes or safety measures were included. Only studies published in English were considered.

Exclusion criteria included narrative reviews, editorials, commentaries, and conference abstracts without full data. Studies that did not involve PRF as an intervention for CRPS, non-human or in vitro studies were also excluded.

References were imported into the review management software, Covidence, for de-duplication and study selection. To minimize the risk for selection bias, articles were independently reviewed by two of three authors (KB and CW) for inclusion or exclusion during both the title/abstract and full-text screening phases. If both authors agreed, their decision was final. In cases of disagreement, a senior author (AN) resolved the conflict.

### 2.3 Data charting

A customized data extraction form was created in Covidence. Data were charted independently by two reviewers (KB and CW) for the following items: study design, sample size, population characteristics, CRPS type, PRF target, outcomes reported, follow-up duration, and adverse events.

### 2.4 Risk of bias

A formal risk of bias assessment was not performed, as this is a scoping review.

### 2.5 Data synthesis

Studies were synthesized descriptively. Data were summarized in tables and figures showing study characteristics, PRF techniques, outcome domains, and safety reporting. Patterns, trends, and gaps in the literature were identified.

## 3 Results

### 3.1 Study selection

The initial database search identified 345 records, corresponding to 344 unique studies, including 149 from Scopus, 84 from PubMed, 57 from PsycINFO, 46 from CINAHL, and 9 from SPORTDiscus. No additional records were identified through citation searching or grey literature sources. After removal of 115 duplicate records, 229 studies remained for title and abstract screening.

During title screening, 217 studies were excluded, primarily because they did not directly evaluate pulsed radiofrequency as an intervention for complex regional pain syndrome. The most common reasons for exclusion included: (1) studies evaluating continuous radiofrequency ablation or other neuromodulation modalities (e.g., spinal cord stimulation, dorsal root ganglion stimulation) without PRF-specific outcomes; (2) studies involving chronic neuropathic pain conditions other than CRPS (e.g., postherpetic neuralgia, radiculopathy, or peripheral neuropathy); (3) review articles, editorials, commentaries, or conference abstracts lacking original patient-level data; and (4) non-human or laboratory-based studies.

**Fig. 1:**
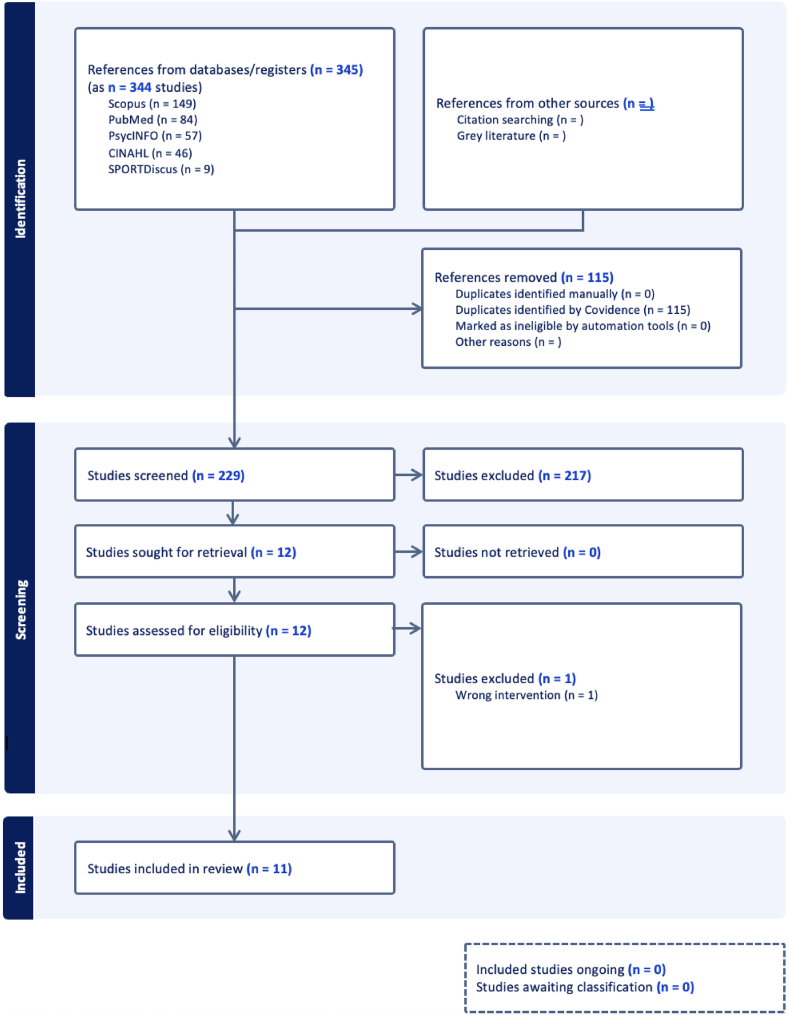
PRISMA flow diagram of study selection for PRF in CRPS.

Twelve studies were sought for full-text retrieval, all of which were successfully obtained and assessed for eligibility. One study was excluded at the full-text stage due to the use of an incorrect intervention (continuous radiofrequency rather than pulsed radiofrequency). Ultimately, 11 studies met the inclusion criteria and were included in this scoping review. Given the limited number of eligible studies identified during screening and the heterogeneity of available evidence, the review was conducted as a scoping review to systematically map the existing literature rather than perform quantitative synthesis. This approach allowed for inclusion of diverse study designs and facilitated identification of key methodological gaps in the current evidence base. A PRISMA flow diagram detailing the study selection process is shown in Fig. 1.

### 3.2 Study characteristics

The 11 included studies comprised primarily case reports and small case series, with a limited number of observational studies; no large-scale randomized controlled trials were identified. Geographically, the included studies were stratified across several countries including South Korea (n = 4), Turkey (n = 3), India (n = 2), Saudi Arabia (n = 1), and Canada (n = 1).

Across all studies, a total of 43 patients were treated with PRF for CRPS, with sample sizes ranging from single-patient reports to small multi-patient cohorts, reflecting the exploratory nature of the existing literature.

The included studies evaluated patients diagnosed with CRPS type I, CRPS type II, or mixed CRPS populations, though several studies did not clearly specify CRPS type I or type II. Diagnostic criteria varied across studies, with some explicitly referencing International Association for the Study of Pain (IASP) or Budapest criteria, while others relied on clinical diagnosis without standardized definitions. In select cases, additional modalities were utilized to supplement initial clinical diagnostic findings, such as three-phase bone scintigraphy, digital infrared thermography, and nerve conduction studies.

Publication years spanned primarily the last two decades, with the oldest study being published in 2008, indicating the relatively novel interest in PRF as a treatment modality for CRPS. Follow-up durations varied considerably, ranging from short-term assessments over weeks to longer-term follow-up extending several months, with inconsistent reporting across studies. A summary of key study characteristics is provided in Table 1.

**Table 1:** Characteristics and outcomes of included studies.

Study (Year)	Country	Design	N	M:F	Mean Age	CRPS Type	CRPS Secondary To	PRF Target	Baseline Pain	Latest Pain	Latest Follow-up
**Akkoc (2008)**	Turkey	Case report	1	0:1	55	NR	Spinal cord injury	Lumbar sympathetic chain	9.5	4	4 m
**Albayrak (2016)**	Turkey	Case series	2	0:2	58.5	I	Stroke	Cervical DRG	9	3	10 m
**Apiliogullari (2015)**	Turkey	Case report	1	0:1	16	I	Poliomyelitis	Lumbar DRG	10	1	6 m
**Chae (2016)**	South Korea	Case report	2	1:1	30.5	I/II	Superficial peroneal nerve	Superficial peroneal nerve	7.5	4.5	4 m
**Choi (2017)**	Saudi Arabia	Case report	1	1:0	43	II	Sciatic nerve	Sciatic nerve	6.5	2.5	8 m
**Djuric (2014)**	Canada	Case series	3	1:2	39.3	I	Traumatic LE injury	Lumbar sympathetic chain	NR	NR	20 m
**Kim (2017)**	South Korea	Retrospective cohort	12	10:2	45.2	NR	Traumatic injury, subsequent surgery	Cervical sympathetic chain	7.75 ± 0.87	4.83 ± 1.19	1 w
**Kumar (2023)**	India	Case series	5	4:1	35.2	NR	Brachial plexus injury	Stellate ganglion	8.6	3.4	3 m
**Oh (2022)**	South Korea	Case report	1	1:0	40	II	Saphenous nerve	Saphenous nerve	7.5	2.5	3 m
**Park (2019)**	South Korea	Retrospective cohort	15	11:4	47.8	NR	Upper extremity (chronic)	Cervical/Thoracic sympathetic ganglion	7.71 ± 0.76/7.80 ± 0.92	3.14 ± 1.06/4.60 ± 1.07	1 w
**Singh Rana (2015)**	India	Case report	1	1:0	34	II	Stretch-induced UE neuropathic injury	Stellate ganglion	8	NR	14 m

**Abbreviations:** CRPS = complex regional pain syndrome; PRF = pulsed radiofrequency; DRG = dorsal root ganglion; UE = upper extremity; LE = lower extremity; NR = not reported; m = months; w = week.

CRPS type (I, II, or I/II) is reported as described by the study authors. NR in the CRPS Type column indicates that classification was not explicitly specified in the original publication. NR in the pain score column indicates that numerical pain values were not reported, although relevant qualitative pain improvement was detailed in the study. For retrospective cohort studies, pain scores are reported as presented in the original study (mean ± standard deviation). In Park et al., values are reported separately for the thoracic and cervical sympathetic ganglion groups.

### 3.3 PRF intervention details

PRF was applied to a diverse range of anatomical targets across the included studies. The most commonly targeted sites were sympathetic structures, including the lumbar and cervical sympathetic chains, the thoracic sympathetic ganglion, and the stellate ganglion. In regard to peripheral nerve targets, studies included the superficial peroneal, sciatic, and saphenous nerves. Two studies applied PRF to the dorsal root ganglion.

Of note, PRF guidance techniques varied across included studies. Several modalities were utilized to supplement PRF intervention including CT, fluoroscopy, as well as ultrasound guidance. Parameters regarding PRF application were also variably reported such as temperature, duration, and voltage. Stimulation protocols were commonly performed prior to PRF application to confirm appropriate needle placement. Sensory stimulation was typically conducted at 50 Hz, and motor stimulation at 2 Hz. Reported sensory thresholds generally ranged between approximately 0.2 and 0.7 V, with motor thresholds up to roughly 1.2 V in select studies.

PRF procedural parameters were reported with variable levels of detail across studies. In all included studies that specified temperature, the electrode tip was maintained at or below 42 °C. Treatment duration most commonly consisted of 120 s per cycle, with reported durations ranging from 120 to 420 s. Several studies administered repeated cycles at the same anatomical level or at adjacent levels. When detailed, PRF current parameters were most commonly delivered at 2 Hz with 20-ms active pulses and voltage settings of approximately 45 V. However, voltage and pulse characteristics were not uniformly reported. These results are summarized in Table 2.

**Table 2:** Summary of PRF intervention parameters and reporting across included studies.

Study (Year)	Diagnostic Criteria	PRF Temperature (°C)	Pulse Frequency (Hz)	Pulse Width (ms)	Voltage (V)	Duration per Cycle (s)	Number of Cycles	Repeated Cycles	Sensory Testing	Motor Testing	Adverse Events	Notes
Akkoc (2008) [16]	NR	42	NR (stimulation at 50 Hz reported, but PRF delivery frequency not specified)	NR	Sensory testing: 0.2-0.5 V (up to 1 V); PRF voltage: NR	240	NR	Yes - applied at L2, L3, and L4	Yes	No	No	PRF performed under CT fluoroscopy guidance using 20G RF needle (150 mm, 10 mm active tip). Sensory stimulation performed at 50 Hz to reproduce concordant pain. PRF pulse width, voltage, frequency, and number of cycles not reported.
Albayrak (2016) [12] – Case 1	Budapest Clinical Criteria	≤42	2	20 ms active pulses (480 ms silent period)	45	120	NR	Yes - applied at C5 and C6 DRG	Yes	No	No	Sensory stimulation prior to PRF: 0.6 V (C5) and 0.7 V (C6); impedance <600 Ω. PRF parameters well specified; number of cycles not reported.
Albayrak (2016) [12] – Case 2	Budapest Clinical Criteria	≤42	2	20 ms active pulses (480 ms silent period)	45	120	NR	Yes - applied at C5 and C6 DRG	Yes	No	No	PRF performed using identical technique to Case 1. Sensory stimulation threshold 0.7 V at both C5 and C6 DRG; impedance <600 Ω. Number of cycles not specified.
Apiliogullari [17] (2015)	Budapest Research Criteria	≤42	2	20 ms active pulses 45 (480 45 ms silent period)	45	120	NR	NR	No	No	NR	Sensory/motor testing not reported; procedure repeated at L4 DRG; pulse width includes active and silent period
Chae [18] (2016) – Case 1	NR	≤42	NR	NR	45	120	3	Yes - repeated at same SPN	Yes	No	No	PRF applied to superficial peroneal nerve (SPN) under ultrasound guidance. Paresthesia elicited at <0.3 mA. Pulse frequency and pulse width not reported.
Chae [18] (2016) – Case 2	NR	≤42	NR	NR	45	120	NR	NR	Yes (US + nerve stimulator-guided SPN localization)	No	No	PRF applied to superficial peroneal nerve (SPN) under ultrasound guidance using identical technique to prior SPN case. Pulse frequency, pulse width, and number of cycles not explicitly stated.
Choi [19] (2017)	IASP Clinical Criteria (CRPS Type II)	42	NR	NR	NR	120	1	4 times	Yes	No	NR	Pulse frequency, pulse width, voltage, motor testing, adverse events not reported
Djuric [20] (2014)	IASP Clinical Criteria	42	2	20	NR	120-180	1 per lesion	2-4 lesions per level (rotation/repositioning)	No	No	NR	Voltage not reported; sensory/motor testing, adverse events not reported; duration varies
Kim [10] (2017)	IASP Clinical Criteria	42	50 (sensory), 2 (motor)	NR	NR	420	1 per level	C6 and C7 levels	Yes	Yes	NR	Pulse width, voltage, adverse events not reported; sensory/motor stimulation used to confirm placement
Kumar [21] (2023)	IASP Clinical Criteria	42	50	NR	Sensory: up to 0.6V; Motor: up to 1.2V	420	1 per lesion	PRF repeated once at same level	Yes	Yes	NR	Pulse width not reported; immediate complications monitored, long-term adverse events not reported
Oh [22] (2022)	NR	42	20	NR	5	120	1 per cycle	2 cycles	Yes	No	No	Pulse width and motor testing not reported; PRF applied after confirming test stimulation; good clinical outcome reported
Park [23] (2019)	IASP Clinical Criteria	42	50 (sensory), 2 (motor)	20	45	360 (TSG), 420 (CSC)	1 per level	TSG: repeated at T2–T3; CSC: repeated at C6–C7	Yes	Yes	NR	PRF applied at multiple levels; sensory/motor stimulation performed; TSG pulse width 20 ms, CSC pulse width not explicitly stated; adverse events not reported.
Singh Rana [24] (2015)	Budapest Clinical Criteria	42	2	20	45	120	2 per location	3 locations at C7	No	Yes (arm/hand paresthesia, vocal cord check)	No	Sensory testing not explicitly reported; procedure well tolerated with long-term follow-up

**Abbreviations:** IASP = International Association for the Study of Pain; NR = not reported; TSG = thoracic sympathetic ganglion; CSC = cervical sympathetic chain; DRG = dorsal root ganglion.

### 3.4 Outcomes reported

Pain score was the primary outcome reported across all included studies. Pain scores were assessed with the VAS (Visual Analog Scale) or with the NRS (Numerical Rating Scale). However, reporting formats varied, with some studies presenting individual case data and others reporting small cohort results. VAS scores originally presented on a 0–100 scale were converted to a 0–10 scale to facilitate consistent comparison. Pain score was acquired in most cases at baseline and at follow-up, status post PRF. However, in select studies, a numerical value was not explicitly obtained at follow-up and alternatively, a qualitative description was provided regarding pain improvement.

Follow-up duration across the studies varied considerably. Follow-up timeframes were recorded from baseline to the latest point of follow-up after PRF. Intervals ranged from as low as 1 week up to the longest interval of 20 months. However, most studies reported timeframes between 3 and 10 months.

Additional outcomes beyond pain score were reported, albeit inconsistently. Uniquely, one retrospective study measured mean inter-limb temperature difference as a quantitative outcome measurement before and after PRF as an indicator of sympathetic blockade and treatment response. Also, other studies descriptively detailed outcomes such as functional improvement, reduction of medication use, and range of motion improvement. However, standardized multidimensional patient-reported outcome instruments were not employed in the included studies.

Importantly, interpretation of pain reduction outcomes is limited by the reliance on subjective pain scales without control groups, making results particularly susceptible to placebo effects and regression to the mean. Additionally, concurrent therapies, including pharmacologic management, physical therapy, or prior interventional procedures, were inconsistently reported and may have contributed to observed improvements.

### 3.5 Adverse events

In all of the included studies, no adverse events were reported. However, some studies did not explicitly address whether adverse events occurred or not.

### 3.6 Evidence gaps and trends

The included literature surrounding PRF for CRPS is comprised of case reports, small case series, and a limited number of retrospective cohort studies. No RCTs or prospective comparative studies were identified.

Methodological heterogeneity is evident across studies. Diagnostic criteria along with CRPS type I or type II were not consistently specified. Additionally, PRF procedural parameters significantly varied with respect to factors such as duration, cycles, stimulation thresholds, etc.

Pain intensity was the consistent primary outcome measure across all studies. However, follow-up intervals varied (Table 1). There also was a lack of utilization regarding validated multidimensional PROM instruments. With respect to adverse event reporting, it was limited and sparsely reported, though studies that did report safety outcomes surrounding PRF, generally noted no complications.

A structured critical appraisal of the included studies, including study design, level of evidence, control group status, and key methodological limitations, is presented in Table 3.

**Table 3:** Critical appraisal of included studies.

Study (Year)	Control Group Present?	Level of Evidence	Key Methodological Limitations
**Akkoc (2008)**	No	V	Single case report with no control group; very limited generalizability, high risk of publication bias, and inability to assess causality or control for placebo effect/concurrent treatments.
**Albayrak (2016)**	No	IV	Very small case series (n = 2) without control group; high risk of selection and publication bias, limited generalizability, and inability to distinguish treatment effect from placebo or concurrent therapies.
**Apiliogullari (2015)**	No	V	Single case report with no control group; highly limited generalizability and high risk of publication bias.
**Chae (2016)**	No	V	Two case reports with no control group; highly limited generalizability and high risk of publication bias.
**Choi (2017)**	No	V	Single case report without control group; extremely limited generalizability, high risk of publication bias, and inability to infer causality or separate treatment effect from placebo response or concurrent therapies.
**Djuric (2014)**	No	IV	Small case series (n = 3) without control group; high risk of selection and publication bias, limited sample size, and inability to control for placebo effect or concurrent therapies.
**Kim (2017)**	No	III	Retrospective design without control group; potential selection bias, limited ability to infer causality, heterogeneity in PRF parameters and targets, and inconsistent reporting of functional outcomes and follow-up duration.
**Kumar (2023)**	No	IV	Small case series without control group; limited sample size, heterogeneity in PRF application parameters and anatomical targets, short and variable follow-up, and inconsistent reporting of functional and quality-of-life outcomes.
**Oh (2022)**	No	V	Single case report without control group; extremely limited generalizability, high risk of publication bias, and inability to infer causality or distinguish treatment effect from placebo response or concurrent therapies.
**Park (2019)**	No	III	Retrospective cohort without control group; potential selection bias, limited ability to infer causality, heterogeneity in PRF targets and parameters, and inconsistent reporting of functional outcomes and follow-up duration.
**Singh Rana (2015)**	No	V	Single case report without control group; highly limited generalizability, high risk of publication bias, and inability to infer causality or control for placebo effect or concurrent treatments.

Overall, the available evidence reflects growing exploration of PRF for CRPS across diverse targets and clinical presentations; though it also underscores the need for standardized diagnostic criteria, uniform reporting of procedural parameters, and structured study designs to better investigate the clinical role of PRF for CRPS.

## 4 Discussion

### 4.1 Summary

In this scoping review, we synthesized the current evidence on PRF for the management of CRPS, a condition known for its complex pathophysiology, heterogenous presentation, and therapeutic challenges. Across the included studies, PRF was frequently associated with reductions in reported pain scores. However, these findings must be interpreted cautiously given the predominance of uncontrolled study designs, small sample sizes, and reliance on subjective outcome measures. The absence of comparator groups limits the ability to attribute observed improvements directly to PRF, as opposed to placebo response, natural disease fluctuation, or the effects of concurrent therapies.

These findings must be interpreted in the context of the fragmented and methodologically heterogeneous literature base. Many studies were limited by small sample sizes, lack of control groups, and inconsistent reporting of key procedural details such as voltage, pulse width, and number of cycles. Furthermore, the diversity in CRPS type I or type II representation and the absence of standardized diagnostic criteria in some studies complicate cross-comparison and generalizability. While PRF appears to offer a favorable safety profile compared with neurodestructive procedures, reporting of adverse events was often incomplete. Among studies that explicitly commented on adverse events, none were reported; however, several studies did not clearly state whether adverse events were assessed or occurred. The absence of reported complications should not be interpreted as definitive evidence of safety, given the small sample sizes and inconsistent reporting of adverse events. This underscores the need for more rigorous safety monitoring in future research.

Taken together, our review suggests that PRF may be a promising adjunct in the multidisciplinary management of CRPS, particularly for patients with refractory pain. However, current evidence is insufficient to establish definitive recommendations regarding optimal treatment parameters, patient selection, or long-term outcomes. High-quality randomized controlled trials with standardized protocols, clear diagnostic criteria, and robust outcome measures are urgently needed to clarify the role of PRF in CRPS care.

### 4.2 Clinical implications

The findings of this review suggest that PRF may represent a safe, minimally invasive option for patients with CRPS who have not responded to conventional therapies. Given its neuromodulatory mechanism and favorable safety profile compared with neurodestructive interventions, PRF can be considered as part of a multimodal treatment plan, particularly in specialized pain management settings. However, the variability in procedural techniques and target selection observed across studies highlights the importance of individualized treatment planning and careful patient selection. Clinicians should interpret the current evidence cautiously, recognizing that long-term efficacy and optimal parameter settings remain uncertain. Until high-quality, standardized trials are available, PRF use in CRPS should be guided by both patient-specific factors and the expertise of the treating interventionalist.

### 4.3 Strengths and limitations

A key strength of this review is its comprehensive and systematic approach to identifying and synthesizing all available evidence on PRF for the treatment of CRPS. We followed established PRISMA guidelines, conducted a broad search across multiple databases, and applied rigorous eligibility criteria to ensure relevance and methodological transparency. By evaluating both efficacy and safety outcomes and exploring potential influences of treatment parameters and CRPS type I or type II, this review provides a balanced and clinically relevant synthesis.

However, several limitations must be acknowledged. Ultimately, only 11 studies met all inclusion criteria, underscoring a substantial evidence gap characterized by limited primary clinical research specifically evaluating PRF for CRPS. This attrition reflects both the narrow scope of the intervention of interest and the overall paucity of rigorously reported clinical studies in this domain, further supporting the appropriateness of a scoping review to map existing evidence and identify areas requiring future investigation. The included studies were also heterogeneous in terms of patient populations, diagnostic criteria, anatomical targets, PRF parameters, and outcome measures, which limited the feasibility of quantitative synthesis and reduced comparability between studies. All studies had small sample sizes, lacked randomization or control groups, and were at risk of bias due to incomplete reporting or short follow-up periods. Adverse event reporting was often sparse or absent, preventing a robust assessment of safety. Additionally, restricting inclusion to English-language publications may have introduced language bias, and the evolving diagnostic frameworks for CRPS may have resulted in variability in case definitions across studies. An additional limitation is the heavy reliance on pain intensity as the primary outcome measure. Functional outcomes, quality of life, and return to activity, arguably more clinically meaningful endpoints in CRPS, were inconsistently reported. Furthermore, follow-up durations varied widely and were often short, limiting assessment of the durability of treatment effects and long-term safety.

### 4.4 Recommendations for future research

Future studies on PRF for CRPS should prioritize high-quality prospective cohort studies or randomized controlled trials with adequate sample sizes, clearly defined diagnostic criteria, and standardized treatment protocols. Consistency in procedural parameters—such as target selection, voltage, pulse duration, and treatment cycles—will be essential to allow for meaningful cross-study comparisons.

### 4.5 Critical appraisal of included evidence

Although a formal risk of bias assessment was not performed in accordance with scoping review methodology, a critical appraisal of the included studies reveals several important limitations that impact interpretation of the findings. The overall level of evidence is low, consisting predominantly of case reports and small case series, with no randomized controlled trials or prospective comparative studies identified (Table 3). As such, the available data are highly susceptible to selection bias, reporting bias, and publication bias, particularly given the tendency for positive outcomes to be preferentially reported in early exploratory literature.

Additionally, all studies lacked control groups, limiting the ability to distinguish treatment effects from the natural history of CRPS, placebo response, or the impact of concurrent therapies. Variability in diagnostic criteria, inconsistent specification of CRPS type I versus type II, and heterogeneity in PRF targets and procedural parameters further reduce internal validity and comparability across studies.

Taken together, while the existing literature suggests potential benefit, the certainty of evidence remains very low, and conclusions regarding efficacy should be interpreted with caution.

### 4.6 Role of PRF in the CRPS treatment algorithm

The studies included in this review suggest that PRF has primarily been utilized as a later-line or adjunctive intervention in patients with refractory CRPS who have not achieved adequate relief with conventional therapies. In most reports, patients had previously undergone combinations of pharmacologic management, physical therapy, and, in some cases, sympathetic nerve blocks prior to PRF application.

PRF was most commonly applied targeting sympathetic structures, dorsal root ganglia, or peripheral nerves, often selected based on the clinical distribution of pain and suspected underlying pathophysiology. This variability reflects the absence of standardized treatment algorithms and highlights the individualized nature of interventional decision-making in CRPS.

Given the current evidence, PRF may be best conceptualized as a minimally invasive neuromodulatory option positioned between diagnostic/therapeutic nerve blocks and more invasive neuromodulation strategies such as spinal cord stimulation or dorsal root ganglion stimulation. However, its precise role remains to be defined through higher-quality comparative studies.

## 5 Conclusion

PRF shows promise as a minimally invasive, neuromodulatory intervention for complex regional pain syndrome, with evidence suggesting potential benefits in pain reduction and, in some cases, functional improvement. However, the current literature is limited by methodological heterogeneity, small sample sizes, and inconsistent reporting, preventing definitive conclusions regarding its efficacy, safety, and optimal use. While PRF may be considered as part of an individualized, multidisciplinary treatment plan for refractory CRPS, its role remains to be clearly defined. Well-designed, adequately powered studies with standardized protocols and long-term follow-up are essential to establish best practices and guide evidence-based clinical decision-making.

## Funding

The authors declare that no funds, grants, or other support were received during the preparation of this manuscript.

## Competing interests

The authors have no relevant financial or non-financial interests to disclose.
