# Supplementary material for: Pulsed radiofrequency for treatment of complex regional pain syndrome: A scoping review
Source: Interv Pain Med. 2026 May 18;5(2):100771. doi: 10.1016/j.inpm.2026.100771 (PMC13213290; doi:10.1016/j.inpm.2026.100771)
Supplement: Multimedia component 1 [file mmc1.docx]

**Appendix – Search Methods:**

**Searches conducted by:** Sarah Wade, MLS, and Emily A. Brennan, MLIS

**PubMed (U.S. National Library of Medicine, National Institutes of Health) search strategy:**

("Complex Regional Pain Syndromes"[Mesh] OR “complex regional”[tiab] OR “complex pain”[ti] OR “chronic regional pain syndrome*”[tiab] OR CRPS[ti] OR “chronic traumatic oedema*”[tiab] OR "Causalgia"[Mesh] OR causalgia[tiab] OR neuroalgodystroph*[tiab] OR “neurovascular dystroph*”[tiab] OR “peripheral trophoneurosis”[tiab] OR “post traumatic complex pain”[tiab] OR “post traumatic algodystroph*”[tiab] OR “post traumatic algoneurodystroph*”[tiab] OR “post traumatic dystroph*”[tiab] OR “post traumatic neurodystroph*”[tiab] OR “post traumatic  osteoporosis”[tiab] OR “reflex neurovascular dystroph*”[tiab] OR “Reflex Sympathetic Dystrophy"[Mesh] OR “reflex sympathetic dystroph*”[tiab] OR RSD[ti] OR “regional pain syndrome*”[tiab] OR “shoulder hand syndrome*”[tiab] OR Sudeck's[tiab] OR Sudecks[tiab] OR sympathalgia[tiab] OR “sympathetic dystroph*"[tiab] OR “sympathetic pain"[tiab] OR “transient migratory osteoporosis”[tiab]) **AND** ("Pulsed Radiofrequency Treatment"[Mesh] OR "pulsed radiofrequenc*"[tiab] OR "pulsed radio frequenc*"[tiab] OR "pulse radiofrequenc*"[tiab] OR "pulse radio frequenc*"[tiab] OR "pulsed radiofrequency"[tiab:~3] OR "pulsed radio frequency"[tiab:~3] OR "pulse radiofrequency"[tiab:~3] OR "pulse radio frequency"[tiab:~3] OR PRF[ti] OR radiofrequenc*[ti] OR neuromodulat*[ti])

- Filters/limits: English
- # of records identified: 84
- Date searched: October 31, 2025

**Scopus (Elsevier) search strategy (line-by-line search):**

**#1: TITLE-ABSTRACT-KEYWORD:** {complex regional} OR “chronic regional pain syndrome” OR “chronic traumatic oedema” OR causalgia OR neuroalgodystroph* OR “neurovascular dystrophy” OR {peripheral trophoneurosis} OR {post traumatic complex pain} OR “post traumatic algodystrophy” OR “post traumatic algoneurodystrophy” OR “post traumatic dystrophy” OR “post traumatic neurodystrophy” OR “post traumatic  osteoporosis” OR “reflex neurovascular dystrophy” OR “reflex sympathetic dystrophy" OR “regional pain syndrome” OR “shoulder hand syndrome” OR Sudeck's OR Sudecks OR sympathalgia OR “sympathetic dystrophy" OR {sympathetic pain} OR “transient migratory osteoporosis”

**#2: TITLE:** {complex pain} OR CRPS OR RSD

**#3:** #1 OR #2

**#4: TITLE-ABSTRACT-KEYWORD:** "pulsed radiofrequency" OR "pulsed radio frequency" OR "pulse radiofrequency" OR "pulse radio frequency”

**#5: TITLE:** PRF OR radiofrequency OR neuromodulat*

**#6:** #4 OR #5

**#7:** #3 AND #6

- Filters/limits: English
- # of records identified: 149
- Date searched: October 31, 2025

**Scopus (Elsevier) search strategy (Advanced Document Search):**

( ( TITLE-ABS-KEY ( {complex regional} OR "chronic regional pain syndrome" OR "chronic traumatic oedema" OR causalgia OR neuroalgodystroph* OR "neurovascular dystrophy" OR {peripheral trophoneurosis} OR {post traumatic complex pain} OR "post traumatic algodystrophy" OR "post traumatic algoneurodystrophy" OR "post traumatic dystrophy" OR "post traumatic neurodystrophy" OR "post traumatic osteoporosis" OR "reflex neurovascular dystrophy" OR "reflex sympathetic dystrophy" OR "regional pain syndrome" OR "shoulder hand syndrome" OR Sudeck's OR Sudecks OR sympathalgia OR "sympathetic dystrophy" OR {sympathetic pain} OR "transient migratory osteoporosis" ) OR TITLE ( {complex pain} OR CRPS OR RSD ) ) ) AND ( ( TITLE-ABS-KEY ( "pulsed radiofrequency" OR "pulsed radio frequency" OR "pulse radiofrequency" OR "pulse radio frequency" ) OR TITLE ( PRF OR radiofrequency OR neuromodulat* ) ) ) AND ( LIMIT-TO ( LANGUAGE , "English" ) )

**APA PsycINFO (EBSCOhost) search strategy:**

(SU "Complex Regional Pain Syndrome (Type I)" OR “complex regional” OR TI “complex pain” OR “chronic regional pain syndrome*” OR TI CRPS OR “chronic traumatic oedema*” OR causalgia OR SU "Neuralgia" OR neuroalgodystroph* OR “neurovascular dystroph*” OR “peripheral trophoneurosis” OR “post traumatic complex pain” OR “post traumatic algodystroph*” OR “post traumatic algoneurodystroph*” OR “post traumatic dystroph*” OR “post traumatic neurodystroph*” OR “post traumatic  osteoporosis” OR “reflex neurovascular dystroph*” OR “reflex sympathetic dystroph*” OR TI RSD OR “regional pain syndrome*” OR “shoulder hand syndrome*” OR Sudeck's OR Sudecks OR sympathalgia OR “sympathetic dystroph*" OR “sympathetic pain" OR “transient migratory osteoporosis”) **AND** (pulsed * radiofrequenc* OR pulsed * radio frequenc* OR pulse * radiofrequenc* OR pulse * radio frequenc* OR TI PRF OR TI radiofrequenc* OR TI neuromodulat*)

- Filters/limits: English
- # of records identified: 57
- Date searched: October 31, 2025

**CINAHL Complete (EBSCOhost) search strategy:**

(MH "Complex Regional Pain Syndromes+" OR “complex regional” OR TI “complex pain” OR “chronic regional pain syndrome*” OR TI CRPS OR “chronic traumatic oedema*” OR MH "Causalgia" OR causalgia OR neuroalgodystroph* OR “neurovascular dystroph*” OR “peripheral trophoneurosis” OR “post traumatic complex pain” OR “post traumatic algodystroph*” OR “post traumatic algoneurodystroph*” OR “post traumatic dystroph*” OR “post traumatic neurodystroph*” OR “post traumatic  osteoporosis” OR “reflex neurovascular dystroph*” OR MH "Reflex Sympathetic Dystrophy" OR “reflex sympathetic dystroph*” OR TI RSD OR “regional pain syndrome*” OR “shoulder hand syndrome*” OR Sudeck' OR Sudecks OR sympathalgia OR “sympathetic dystroph*" OR “sympathetic pain" OR “transient migratory osteoporosis”) **AND** (pulsed * radiofrequenc* OR pulsed * radio frequenc* OR pulse * radiofrequenc* OR pulse * radio frequenc* OR TI PRF OR TI radiofrequenc* OR MH "Neurotransmitters" OR TI neuromodulat*)

- Filters/limits: English
- Expanders: apply equivalent subjects
- Search mode: proximity
- Date searched: October 31, 2025
- # of records identified: 46

**SPORTDiscus (EBSCOhost) search strategy:**

(DE "Complex Regional Pain Syndromes" OR “complex regional” OR TI “complex pain” OR “chronic regional pain syndrome*” OR TI CRPS OR “chronic traumatic oedema*” OR causalgia OR neuroalgodystroph* OR “neurovascular dystroph*” OR “peripheral trophoneurosis” OR “post traumatic complex pain” OR “post traumatic algodystroph*” OR “post traumatic algoneurodystroph*” OR “post traumatic dystroph*” OR “post traumatic neurodystroph*” OR “post traumatic  osteoporosis” OR “reflex neurovascular dystroph*” OR “reflex sympathetic dystroph*” OR TI RSD OR “regional pain syndrome*” OR “shoulder hand syndrome*” OR Sudeck' OR Sudecks OR sympathalgia OR “sympathetic dystroph*" OR “sympathetic pain" OR “transient migratory osteoporosis”) **AND** (pulsed radiofrequenc* OR pulsed radio frequenc* OR pulse radiofrequenc* OR pulse radio frequenc* OR TI PRF OR TI radiofrequenc* OR TI neuromodulat*)

- Filters/limits: English
- Date searched: October 31, 2025
- # of records identified: 9

**ClinicalTrials.gov (U.S. National Library of Medicine, National Center for Biotechnology Information) search strategy:**

**Condition/disease:** complex regional pain syndromes

**Intervention/treatment:** pulsed radiofrequency

**Study results:** with results

- # of records identified: 0
- Date searched: October 31, 2025

**International Clinical Trials Registry Platform (ICTRP) (World Health Organization) search strategy:**

complex regional pain syndromes AND pulsed radiofrequency

- Filters/limits: none
- # of records identified: 0
- Date searched: October 31, 2025
